# Chilli thrips oviposition behavior: a comparative study among strawberry cultivars

**DOI:** 10.1093/ee/nvaf073

**Published:** 2025-07-10

**Authors:** Lovely Adhikary, Hugh A Smith, Vance M Whitaker, Sriyanka Lahiri

**Affiliations:** Gulf Coast Research and Education Center, Institute of Food and Agricultural Sciences, University of Florida, 14625 CR 672, Wimauma, FL 33598, USA; Gulf Coast Research and Education Center, Institute of Food and Agricultural Sciences, University of Florida, 14625 CR 672, Wimauma, FL 33598, USA; Gulf Coast Research and Education Center, Institute of Food and Agricultural Sciences, University of Florida, 14625 CR 672, Wimauma, FL 33598, USA; Gulf Coast Research and Education Center, Institute of Food and Agricultural Sciences, University of Florida, 14625 CR 672, Wimauma, FL 33598, USA

**Keywords:** cultivar susceptibility, insect–plant interactions, oviposition preference, thrips management strategies

## Abstract

Florida is the second-highest producer of strawberries in the USA. Chilli thrips *Scirtothrips dorsalis* Hood (Thysanoptera: Thripidae) have become a major pest in Florida strawberries following its establishment in the USA after 2005. Insecticide application is the leading management approach for *S. dorsalis*. However, this pest demonstrated the tendency to develop resistance to a broad range of active ingredients. Host–plant resistance (HPR) may contribute to the management of this pest, yet mechanisms of HPR, including antixenosis and antibiosis against *S. dorsalis* in strawberries, are not well studied. Therefore, this study aimed to evaluate the preference of *S. dorsalis* to select strawberry cultivars as oviposition sites that resulted in a successful egg eclosion. Seven commercial strawberry cultivars namely, “Florida Brilliance,” Florida Medallion “FL 16.30-128,” Sweet Sensation “Florida 127,” Florida Pearl “FL 16.78-109,” “Strawberry Festival,” “Florida Beauty,” and “Florida Radiance” were evaluated as hosts in an oviposition choice test. Greenhouse potted strawberry plants were infested with 1- to 3-d-old adult *S. dorsalis* females. The cultivars used as treatment were replicated five times, and the study was repeated twice. Among the seven cultivars, Florida Pearl “FL 16.78-109,” “Florida Beauty,” and “Strawberry Festival” had a higher number of eggs in the leaf tissue compared with other tested cultivars. However, the egg hatching percentage did not demonstrate a specific trend. The results showed that *S. dorsalis* has a strong oviposition preference for certain strawberry cultivars over others, and this information may be incorporated into HPR for managing *S. dorsalis* in strawberries.

## Introduction

Strawberry, *Fragaria* X *ananassa* Duchesne (Rosales: Rosaceae), is one of the prominent horticultural crops in Florida. Strawberries are cultivated on around 6,555 ha (16,200 A) of farmland in central and south Florida, generating more than $540 million in revenue (US Strawberry Industry 2024). Unlike other parts of the USA, strawberries in Florida are grown in winter on double-pressed beds in open fields (Whitaker et al., 2024). The open-field strawberry plants can be infested by several arthropod pests, including a variety of thrips species (Thysanoptera: Thripidae), mites (Arachnida: Acari), and aphids (Hemiptera: Aphididae) (Lahiri et al., 2022). Among these pests, chilli thrips *Scirtothrips dorsalis* Hood (Thysanoptera: Thripidae) has become the major pest of strawberries in Florida. *Scirtothrips dorsalis* females prefer to feed and oviposit on young plant tissue (Panthi and Renkema, 2020). Their feeding results in the darkening of leaf veins and petioles. Extensive infestation leads to upward curling, hardening of leaf tissue, bronzing, and cracking of developing fruits (Panthi and Renkema, 2020). *Scirtothrips dorsalis* is a polyphagous pest and is difficult to control because of its short lifecycle, high reproduction rate, and cryptic behavior (Kumar et al., 2013). Additionally, like other thrips species, *S. dorsalis* lays eggs inside the plant tissue, which are difficult to manage using contact ovicides. Primarily, this pest is managed by the foliar application of systemic insecticides (Seal and Kumar, 2010). However, this approach can lead to the development of insecticide resistance (Reddy et al., 1992, Vanisree et al., 2011). For this reason, it is important to evaluate host plant resistance as a control option for *S. dorsalis*. Resistant cultivars can influence the feeding, oviposition, and larval development of the pest on the host plant (Stout and Davis, 2009). Host plant resistance to insects affects the insect’s behavior or physiology (antixenosis or antibiosis, respectively). Host selection for oviposition is important among herbivores, but the preference behavior differs with the host plant (Fu et al., 2019). Thrips use visual and olfactory cues for host orientation and selection (Teulon et al., 1999). The choice of a host for oviposition depends on behavioral, physiological, and ecological factors influencing the herbivores and their host plants (Singer, 1986). These selections or preferences by ovipositing females influence the survival ability and growth of the offspring on the host plants (Thompson, 1988). This aligns with the theory about the ability of adult females to make prime choices for their offspring, known as “mother knows best hypothesis” (Weber et al., 2020).

Previous studies with western flower thrips, *Frankliniella occidentalis* (Pergande) (Thysanoptera: Thripidae), have shown reduced reproduction rates and longer developmental time on resistant genotypes of cucumbers, *Cucumis sativus* L. (Cucurbitaceae) (Soria and Mollema, 1995). Another study with *F. occidentalis* has shown that the strawberry cultivar “Camarosa” has a higher oviposition and egg hatch compared with the strawberry cultivars “Albion” and “Camino Real” (Rahman et al., 2010). The strawberry leaf beetle, *Galerucella tenella* (L.) (Coleoptera: Chrysomelidae), showed a clear oviposition preference for host plants wild strawberry (*Fragaria vesca* L.) and refrained from laying eggs on the accessions holding higher antibiosis, which can reduce larval performance and survival (Weber et al., 2020). Egg hatching also depends on various factors such as plant age (van Haperen et al., 2019), age of the eggs, photoperiod, and light intensity to which the plant and the eggs were exposed (Blackmer et al., 2002).

*Scirtothrips dorsalis* is known to attack strawberries at a very young stage (Panthi and Renkema, 2020) and tends to remain on initially infested plants (Panthi et al., 2021). The number of eggs laid on a plant is important for population build-up in the field. The varietal difference in host plant suitability for the oviposition preference of *S. dorsalis* has not been studied before. Therefore, there were two objectives of this study: (1) to understand the preference of *S. dorsalis* females for oviposition on various commercial strawberry cultivars and (2) to evaluate the egg hatching percentage among these cultivars. The null hypothesis was that *S. dorsalis* females do not differentiate among various strawberry cultivars for oviposition, and all cultivars would support a similar egg hatching percentage. The alternate hypothesis was that some strawberry cultivars would have more *S. dorsalis* eggs and egg hatching percentage than other cultivars. The results of this study would assist in the identification of potential strawberry cultivars that can reduce the *S. dorsalis* population in the field.

## Materials and Methods

### Laboratory colony of S. dorsalis

The laboratory colony of *S. dorsalis* was reared on upland cotton plants, *Gossypium hirsutum* L. (Malvaceae) (MRC 270 Organic Cotton seeds; TX, USA). The colony was maintained in an environmentally controlled growth room (at 27 ± 5 °C, 60 ± 5% RH, 16:8 L:D photophase) at the UF/IFAS Gulf Coast Research and Education Center (GCREC, Wimauma, FL) (27.7604, −82.2275).

### Strawberry Plant Husbandry

Bare-root transplants of strawberry cultivars namely “Florida Brilliance” (US Patent PP30,564, Whitaker et al., 2019); “Florida Medallion FL16.30-128” (US Patent PP33,451), referred to as “Florida Medallion” hereafter; “Sweet Sensation Florida127” (US Patent PP25,574, Whitaker et al. 2015), referred to as “Sweet Sensation” hereafter; “Florida Pearl FL16.78-109” (US Patent PP 33,477, Whitaker et al., 2023), referred to as “Florida Pearl 109” hereafter; “Strawberry Festival” (US Patent PP14,739, Chandler et al., 2000), “Florida Radiance” (US Patent PP 20,363, Whitaker et al., 2013), and “Florida Beauty” (US Patent PP30,385, Whitaker et al., 2017) were acquired from a commercial nursery (Crown Nursery LLC, CA, USA). The transplants were planted in pots (10.2 cm × 8.9 cm, Greenhouse Megastore, IL, USA) with potting soil (BWI Pro-Mix 2.8 cf; BWI Companies). Plants were kept inside a greenhouse at GCREC with a temperature of 25 ± 5 °C and 75 ± 5% relative humidity (HOBO U23 Prov2; Onset Computer Corporation, MA, USA) and a natural photoperiod. After 15 d, when the plants had at least 3 to 4 trifoliates, they were transferred into screened cages (0.914 m length × 0.762 m width × 0.609 m height).

### Experimental Design

The oviposition bioassay was conducted to evaluate the preference of adult *S. dorsalis* among seven commercial strawberry cultivars mentioned above. One potted strawberry plant from each of these 7 cultivars was arranged randomly inside cages for the experiment (Fig. 5). Each cage was considered as one replication. The experiment was conducted with a randomized complete block design with five replications (five cages). All plants were at a similar growth stage and free from prior infestation, and 1- to 3-d-old adult *S. dorsalis* females (15 per plant × 7 cultivars = 105 adult females per cage) were aspirated into a micropipette. The pipettes were left open to release the *S. dorsalis* in the middle of the cage, which allowed free movement and uninfluenced host selection. The cages were maintained in a controlled greenhouse environment with temperature and humidity consistent across the replicates. Since the adult *S. dorsalis* was reared on cotton plants during their immature stages, they were given a few extra days to adjust to the new host plant. The signs of feeding damage were noticed after a week, and after 3 wk of inoculation, young fully open trifoliates were harvested in a Ziplock bag (Ziplock SC Johnson, Racine, WI) and brought back to the lab. All the leaves were taken out and cleaned with a thick paintbrush so that there were no thrips life stages other than eggs left in the leaves. Three leaves from each of the plants were selected randomly to boil, stain with dye, and count the eggs. Another set of three leaves from the same plant was randomly selected and kept separately in a small petri dish (5 cm diameter, Fisherbrand, Suwanee, GA) provisioned with a moist filter paper (Whatman qualitative filter paper, Sigma-Aldrich, MO, USA) for egg eclosion. The data collection was done once per cultivar (treatment) per cage (replication). The sample size was *N* = 5 per treatment group, that is 35 total observations.

**Fig. 1.**
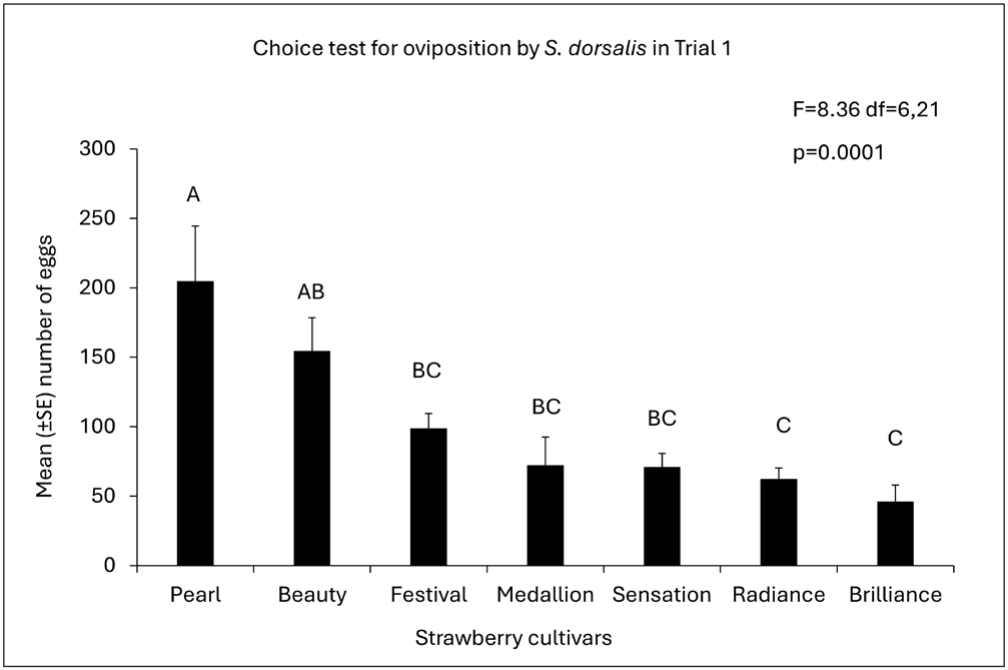
Mean (±SE) of the number of *S. dorsalis* eggs among strawberry cultivars in Trial 1. *P*-values of overall ANOVA obtained by Proc GLIMMIX (SAS on Demand for Academics). Different letters indicate a significant difference among cultivars (*P* < 0.05, post hoc Tukey’s HSD test).

The experiment was repeated twice and referred to as trial 1 and trial 2. The second independent trial was conducted under similar conditions using new sets of plants and insects to assess the consistency of results across time. Data from each trial were analyzed separately to account for any variation and to assess the consistency of cultivar effects over time.

### Leaf Staining Process

The leaf boiling and staining process was performed within a chemical Fume hood (Isolator, Jamestown Metal Products, NY, USA) following the process described in Shrestha et al. (2012). The hood was set up at 39.62 mt per min face velocity at the time of use. A 100 ml boiling solution was made up of 25 ml of glacial acetic acid (Consolidated Chemicals and Solvents, LLC, PA, USA), 25 ml of 10% lactic acid (LD Carlson Company, OH, USA), and 50 ml of 95% ethanol (Fisher Scientific, NH, USA). The leaves were boiled in the boiling solution for 25 to 30 min until the leaves turned completely white. The leaves were then boiled in lactophenol acid fuchsine solution containing 10% lactic acid, 50% glycerin (HACH LANGE GmbH Dusseldorf, Germany), distilled water, saturated phenol (Sigma-Aldrich, MO, USA), and 0.12 grams acid fuchsine (Thermo Fisher, MA, USA) for 4 to 5 min. The excess stain was washed from the leaflets with warm DI water. Stained eggs on the leaflets were observed and counted under a stereomicroscope (Stemi 508, Carl Zeiss, Germany) with 40× magnification (Fig. 6).

**Fig. 2.**
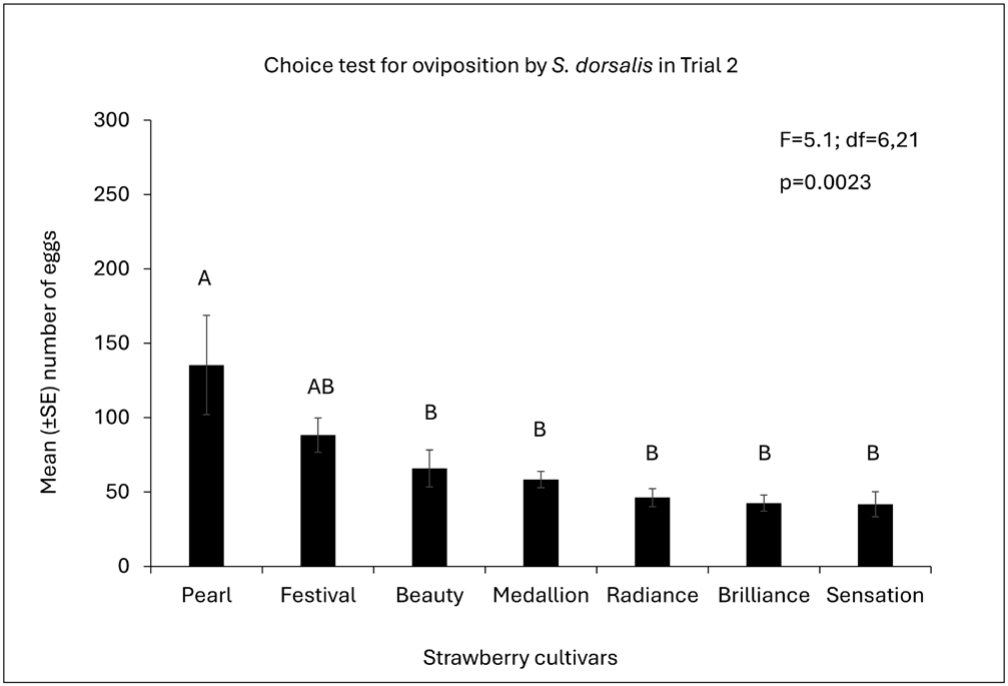
Mean (±SE) of the number of *S. dorsalis* eggs among strawberry cultivars in Trial 2. *P*-values of overall ANOVA obtained by Proc GLIMMIX (SAS on Demand for Academics). Different letters indicate a significant difference among cultivars (*P* < 0.05, post hoc Tukey’s HSD test).

**Fig. 3.**
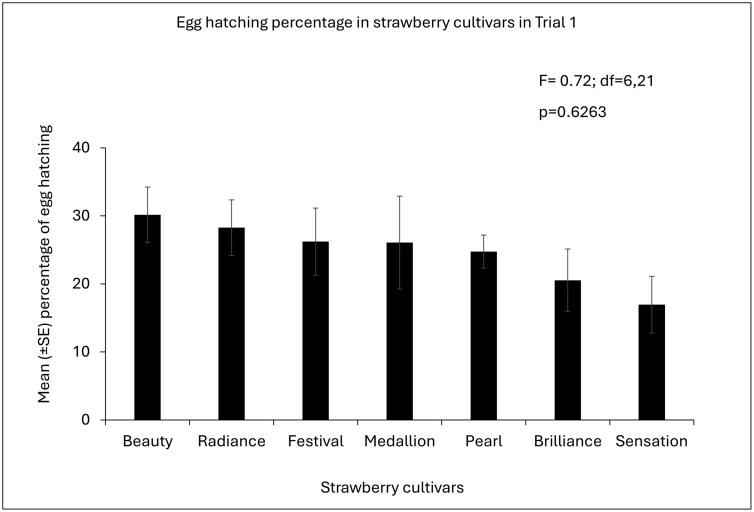
Mean (±SE) percentage of egg eclosion of *S. dorsalis* among strawberry cultivars in Trial 1. *P*-values of overall ANOVA obtained by Proc GLIMMIX (SAS on Demand for Academics). Different letters indicate a significant difference among cultivars (*P* < 0.05, post hoc Tukey’s HSD test).

**Fig. 4.**
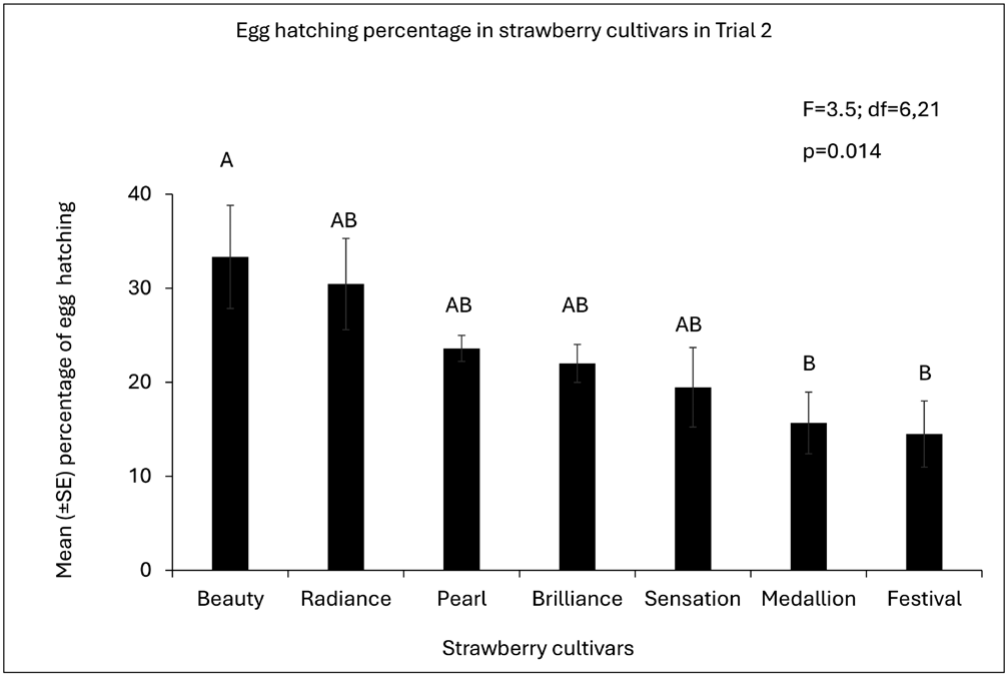
Mean (±SE) percentage of egg eclosion of *S. dorsalis* among strawberry cultivars in Trial 2. *P*-values of overall ANOVA obtained by Proc GLIMMIX (SAS on Demand for Academics). Different letters indicate a significant difference among cultivars (*P* < 0.05, post hoc Tukey’s HSD test).

**Fig. 5.**
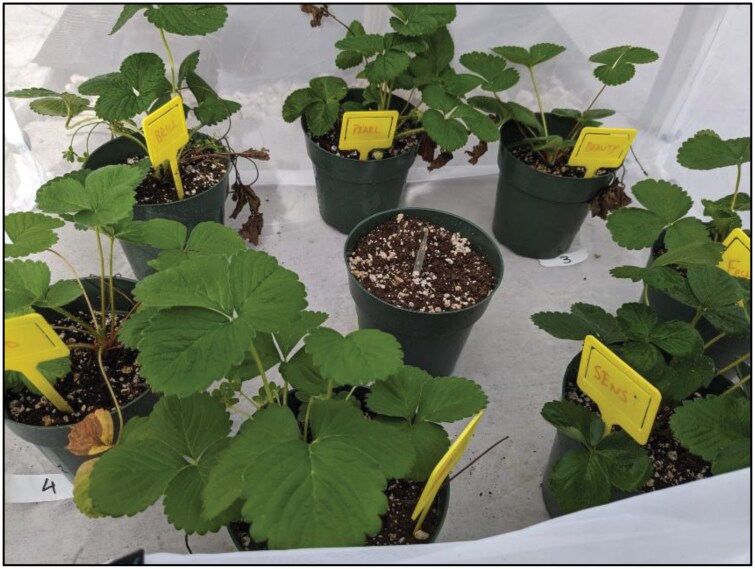
Randomly arranged potted strawberry cultivars inside a cage during the experiment. The pot in the middle has an open micro pipette to release the chilli thrips inside the cage.

**Fig. 6.**
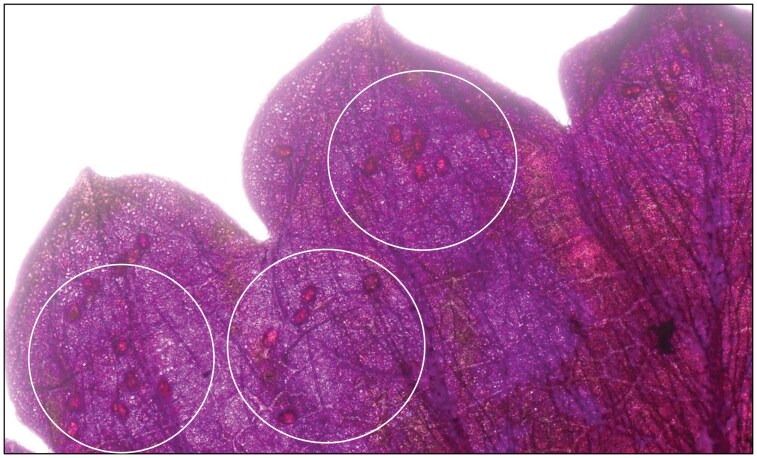
*Scirtothrips dorsalis* eggs are seen as light red grains, highlighted in this picture by circles, inside a stained strawberry leaf.

For egg eclosion, the leaves were checked every day under the microscope, and any larvae hatching from the eggs were counted and removed with a fine brush. After 7 d, these leaves were also boiled and stained to count the number of eggs. The hatching percentage from each cultivar was calculated using the formula:

**Egg hatching rate = no. of larvae/no. of eggs × 100** (Rahman et al., 2010).

### Data Analysis

The data were analyzed using the SAS OnDemand for Academics web platform (SAS Institute Inc., Cary, NC, USA). The normality of residuals was tested using the Shapiro–Wilk test (Proc Univariate). The cultivars as treatments were considered as fixed effects. Replications and the interaction between cultivar and replication were considered as random effects to assess the variability across the cage. Also, replication and its interaction with cultivars were not the primary objective or interest but represented as sources of random variability in the model, hence treated as random effects. This approach produces more accurate and reliable estimates of cultivar influence on oviposition and egg hatching. A generalized linear mixed model was used to understand the effect of cultivars on the oviposition and egg hatching (PROC GLIMMIX). Tukey’s Honestly Significant Difference test was performed as a post-hoc test, α = 0.05, on the significant models.

## Results

Cultivars as treatments significantly influenced the oviposition preference of *S. dorsalis*. In trial 1, the mean number of eggs from trifoliates significantly differed among the cultivars (*F* = 8.36; df = 6,21; *P* = 0.0001; Fig. 1). “Florida Pearl 109” had a significantly higher number of eggs than most cultivars, other than “Florida Beauty.” On the other hand “Florida Brilliance” and “Florida Radiance” had the lowest number of eggs among the cultivars. In Trial 2, the cultivars followed the same trend as in Trial 1, with “Florida Pearl 109” having the highest number of eggs, followed by “Strawberry Festival” (*F* = 5.1, df = 6,21; *P* = 0.0023; Fig. 2).

In Trial 1, the egg eclosion rate among the cultivars did not differ (F = 0.72, df = 6,21; p = 0.6263) (Fig. 3). However, in Trial 2, “Florida Beauty” had a significantly higher egg eclosion percentage than “Strawberry Festival” and “Florida Medallion™” (F = 3.5, df = 6,21; p = 0.014; Fig. 3). Other cultivars namely “Florida Brilliance,” “Florida Pearl 109™,” “Florida Radiance,” and “Sweet Sensation ®” did not differ significantly in egg eclosion percentage from “Florida Beauty” (Fig. 4) (Supplemental table).

## Discussion

Based on the findings of this study, *S. dorsalis* prefers to lay their eggs in the leaves of certain strawberry cultivars over others, but once the eggs are laid, the percentage of successful egg eclosion may remain unaffected by strawberry cultivars. Therefore, there is clear evidence of a preference for an oviposition host in strawberries, where an avoidance of oviposition emerged as the impact on the host selection behavior of *S. dorsalis*. In both trials, *S. dorsalis* showed a preference for “Florida Pearl 109” as a host for oviposition. However, the egg eclosion did not differ among the cultivars in trial 1.

Many thrips species deposit their eggs inside the plant tissue, which is beyond the reach of most biocontrol agents or pesticides. Understanding the host plant characteristics of these cultivars, which influence the oviposition and egg eclosion percentage, can be critical for managing this pest in strawberries. Host selection for oviposition by insects depends on various factors, and this is a complicated process to understand how females select a host plant to deposit eggs  (Potter et al., 2012) . Oviposition in herbivores can be influenced by plant characteristics (Horner and Abrahamson 1992), such as water content, nutrients, and the presence of secondary metabolites (Miller and Strickler, 1984) also the physical and chemical components of the host plant, the microhabitats present on the host plant, and the intensity of infestation (Jaenike, 1978). Studies have shown that polyphagous insects select a host plant for oviposition earlier in their search than monophagous insects. The selection is usually based on the suitability of the host plant for larval development, high fecundity, or fluctuation in the availability of the host plants (Jaenike, 1978).

The results from this study revealed that the strawberry cultivar “Florida Pearl 109” was more suitable as an oviposition host for *S. dorsalis* than other cultivars. This difference in preference could be due to the inherent characteristics of this cultivar. *Scirtothrips dorsalis* predominantly feed on the leaf or vegetative part of the plant instead of flowers, so strawberry leaf nutritional components and physical characteristics can be important factors for their preferences. As this study was a follow-up experiment of a recent field study (Adhikary et al., 2025), the analysis of plant or leaf morphological characteristics or nutritional composition was beyond the scope of this experiment; however, these plant characteristics are important for host selection. For example, the strawberry cultivar “Sweet Charlie” was found to be more resistant to *Tetranychus urticae* Koch (Acari: Tetranychidae) than “Camarosa” due to higher concentrations of total phenols, amino acids, and a higher density of leaf trichomes (Afifi et al., 2010). Another study found that strawberry plants were more resistant to *T. urticae* at the fruiting stage when the concentration of methyl salicylate in the plant increased significantly (Hamilton-Kemp et al., 1988). Strawberry sugar content and monoterpenoid volatile linalool also influenced the oviposition preference of *Drosophila suzukii* (Drosophilidae: Diptera) (Baena et al., 2022). Other plants from the Rosaceae family also showed resistance due to the presence of specific leaf chemicals. For example, the apple cultivar “Florina” was also found to be resistant against the rosy apple aphid *Dysaphis plantaginea* (Passeriini) (Hemiptera: Aphididae) due to the presence of foliar phenolic composition (Rat-Morris 1993). Cultivars of *Capsicum annuum* with higher concentrations of long-chain alkenes showed considerable susceptibility to western flower thrips *F. occidentalis* (Macel et al. 2020). On the other hand, leaf properties such as acyclic diterpene glycosides were present in *F. occidentalis* resistant cultivars of *C. annuum* (Macel et al. 2019).

In this experiment, “Florida Pearl 109,” “Florida Beauty,” and “Strawberry Festival” had more eggs deposited on the leaves by *S. dorsalis*. On the contrary, “Florida Brilliance” and “Florida Radiance” had the lowest number of eggs laid by the adult females. Physical characteristics of plants, such as leaf trichomes, can also be important for insects to select a plant as a host. The lower density of trichomes on the leaf can increase susceptibility. For example, *Heliothrips haemorrhoidalis* (Bouche) (Thysanoptera: Thripidae) severely impacted the mature leaves of Rhododendron (*Rhododendron ferrugineum L*.) (Ericaceae: Ericales) with reduced trichome density (Scott-Brown et al., 2016). Soybean thrips *Neohydatothrips variabilis* (Beach) (Thysanoptera: Thripidae) also showed a lower preference for the soybean cultivars with denser trichomes on the leaf (Zhou et al., 2020). However, the opposite result was found, where trichome density did not influence the *F. occidentalis* resistance in tomatoes (Bac-Molenaar et al., 2019). The difference in the number of eggs laid by adult *S. dorsalis* on different cultivars can be due to the presence of antifeedants or deterrents, coupled with the absence of proper nutrients, which reduce the food intake and impair the egg production ability (Soria and Mollema, 1995, Maharijaya et al., 2012).

The egg eclosion rate differed among the cultivars in trial 2, and “Florida Beauty” had a significantly higher number of larvae than “Strawberry Festival” and “Florida Medallion.” Previous studies have shown that resistance against thrips can change with the age of the plant. For example, larval emergence significantly increases in older leaves of *Capsicum annuum* compared with younger leaves (van Haperen et al., 2019). However, in our study, all the plants were in the same growth stage of their development, and the difference in hatching rates could have been due to the plants’ nutritional factors (Maharijaya et al., 2012). The resistance or susceptibility of the tested strawberry cultivars was due to their suitability for oviposition and feeding by *S. dorsalis* adult females.

To create a sustainable management system for *S. dorsalis*, the inclusion of cultivars that support a lower number of eggs can reduce or delay population development in the field and ultimately reduce chemical pesticide applications.

Future research should explore the additional aspects of host plant resistance to build a more comprehensive understanding of plant–insect interactions. The suitability of cultivars for the development and survival of the immature stages could be a logical next step. The cultivars that are equally selected for oviposition may differ in their ability to support larval development, pupation, and adult emergence, revealing possible antibiosis or postovipositional resistance mechanisms. Future experiments could also include no-choice tests that follow the entire life cycle, recording the survival rates, developmental times, and fecundity across the cultivars.

Additionally, the integration of plant physiological metrics, for example secondary metabolites, volatile organic compounds, and leaf nutritional content, could provide valuable insights about the plant characteristics, driving the preference and performance of *S. dorsalis*. Altogether, these studies would help identify the cultivars that are less preferred as oviposition hosts and less suitable for larval development, contributing to the establishment of integrated pest management strategies.

## Supplementary material

Supplementary material is available at *Environmental Entomology* online.

nvaf073_Supplementary_Material
