# Supplementary material for: Chilli thrips oviposition behavior: a comparative study among strawberry cultivars
Source: Environ Entomol. 2025 Jul 10;54(5):1088–95. doi: 10.1093/ee/nvaf073 (PMC12543318; doi:10.1093/ee/nvaf073)
Supplement: nvaf073_Supplementary_Material [file nvaf073_supplementary_material.docx]

| **Experiment** |  |  |  |  |  |  |  |
| --- | --- | --- | --- | --- | --- | --- | --- |
|  | **Effect** | **Type** | **DF** | **F value** | **P value** | **Covariance** | **SE** |
| Oviposition trial 1 | Cultivar | Fixed | 6, 21 | 8.36 | 0.0001 | ---- | ---- |
|  | Replication | Random | ---- | ---- | ---- | 52.9529 | 115.16 |
|  | Rep*Cult | Random | ---- | ---- | ---- | 0 | . |
|  |  |  |  |  |  |  |  |
| Oviposition trial 2 | Cultivar | Fixed | 6, 21 | 5.1 | 0.0023 | ---- | ---- |
|  | Replication | Random | ---- | ---- | ---- | 0 | . |
|  | Rep*Cult | Random | ---- | ---- | ---- | 1120.00 | 299.59 |
|  |  |  |  |  |  |  |  |
| Hatching percentage trial 1 | Cultivar | Fixed | 6, 21 | 0.72 | 0.6263 | ---- | ---- |
|  | Replication | Random | ---- | ---- | ---- | 29.2612 | 29.5609 |
|  | Rep*Cult | Random | ---- | ---- | ---- | 84.7351 | 24.7468 |
|  |  |  |  |  |  |  |  |
| Hatching percentage trial 2 | Cultivar | Fixed | 6, 21 | 3.5 | 0.014 | ---- | --- |
|  | Replication | Random | ---- | ---- | ---- | 3.24 | 9.6441 |
|  | Rep*Cult | Random | ---- | ---- | ---- | 67.5734 | 19.7914 |
|  |  |  |  |  |  |  |  |

Supplemental Table: F values and P values of the fixed effects and estimated covariance and their corresponding standard errors for the random effects generated through the PROC GLIMMIX on SAS OnDemand for Academics. The estimates of covariance of the random effects provide insight into the magnitude of variability associated with the random factor in the model, also supporting more accurate tests of the fixed effects of the cultivars.
